# An Updated Review of SARS-CoV-2 Vaccines and the Importance of Effective Vaccination Programs in Pandemic Times

**DOI:** 10.3390/vaccines9050433

**Published:** 2021-04-27

**Authors:** Cielo García-Montero, Oscar Fraile-Martínez, Coral Bravo, Diego Torres-Carranza, Lara Sanchez-Trujillo, Ana M. Gómez-Lahoz, Luis G. Guijarro, Natalio García-Honduvilla, Angel Asúnsolo, Julia Bujan, Jorge Monserrat, Encarnación Serrano, Melchor Álvarez-Mon, Juan A De León-Luis, Miguel A. Álvarez-Mon, Miguel A. Ortega

**Affiliations:** 1 Department of Medicine and Medical Specialities, Faculty of Medicine and Health Sciences, University of Alcalá, 28801 Alcalá de Henares, Spain; cielogar@ucm.es (C.G.-M.); oscar.fraile@edu.uah.es (O.F.-M.); lstrujillo@salud.madrid.org (L.S.-T.); anam.gomez@uah.es (A.M.G.-L.); natalio.garcia@uah.es (N.G.-H.); mjulia.bujan@uah.es (J.B.); jorge.monserrat@uah.es (J.M.); mademons@uah.es (M.Á.-M.); maalvarez.gonzalez@salud.madrid.org (M.A.Á.-M.); miguelangel.ortega@edu.uah.es (M.A.O.); 2 Department of Public and Maternal and Child Health, School of Medicine, Complutense University of Madrid, 28040 Madrid, Spain; coral.bravo@salud.madrid.org; 3 Department of Obstetrics and Gynecology, University Hospital Gregorio Marañón, 28009 Madrid, Spain; 4 Health Research Institute Gregorio Marañón, 28009 Madrid, Spain; 5 First of May Health Centre, Health Area I, Rivas Vaciamadrid, 28521 Madrid, Spain; dtorresc@salud.madrid.org; 6 Service of Pediatric, Hospital Universitario Principe de Asturias, 28801 Alcalá de Henares, Spain; 7 Unit of Biochemistry and Molecular Biology (CIBEREHD), Department of System Biology, University of Alcalá, 28801 Alcalá de Henares, Spain; luis.gonzalez@uah.es; 8 Ramón y Cajal Institute of Sanitary Research (IRYCIS), 28034 Madrid, Spain; angel.asunsolo@uah.es; 9 Department of Surgery, Medical and Social Sciences, Faculty of Medicine and Health Sciences, University of Alcalá, 28801 Alcala de Henares, Spain; 10 Los fresnos of Health Centre, Health Area III, Torrejon de Ardoz, 28850 Madrid, Spain; encarnacion.serrano@uah.es; 11 Immune System Diseases-Rheumatology, Oncology Service an Internal Medicine, University Hospital Príncipe de Asturias, (CIBEREHD), 28806 Alcalá de Henares, Spain; 12 Department of Psychiatry and Medical Psychology, Hospital Universitario Infanta Leonor, 28031 Madrid, Spain; 13 Cancer Registry and Pathology Department, Hospital Universitario Principe de Asturias, 28806 Alcalá de Henares, Spain

**Keywords:** SARS-CoV-2, coronavirus, COVID-19, vaccination programs, vaccine acceptance, vaccine hesitancy, pandemic management, mRNA vaccines, adenovirus-based vaccines, inactivated virus vaccines

## Abstract

Since the worldwide COVID-19 pandemic was declared a year ago, the search for vaccines has become the top priority in order to restore normalcy after 2.5 million deaths worldwide, overloaded sanitary systems, and a huge economic burden. Vaccine development has represented a step towards the desired herd immunity in a short period of time, owing to a high level of investment, the focus of researchers, and the urge for the authorization of the faster administration of vaccines. Nevertheless, this objective may only be achieved by pursuing effective strategies and policies in various countries worldwide. In the present review, some aspects involved in accomplishing a successful vaccination program are addressed, in addition to the importance of vaccination in a pandemic in the face of unwillingness, conspiracy theories, or a lack of information among the public. Moreover, we provide some updated points related to the landscape of the clinical development of vaccine candidates, specifically, the top five vaccines that are already being assessed in Phase IV clinical trials (BNT162b2, mRNA-1273, AZD1222, Ad26.COV2.S, and CoronaVac).

## 1. Introduction

Coronavirus disease 2019 (COVID-19) is a complex disease caused by the etiological agent SARS-CoV-2, the novel *Betacoronavirus* first detected in Wuhan, China [1]. COVID-19 has been the largest pandemic ever experienced in the 21st century, having caused, at the time this paper was written (late March 2021), 123.5 million reported cases and 2.7 million deaths worldwide [2]. SARS-CoV-2 has the ability to spread easily and rapidly, overloading sanitary systems and clinical management, and is considered an unprecedented public health concern [3]. Consequently, tough but necessary measures were implemented, including obligated social distancing with important changes to people’s lifestyles. Despite the actions taken, SARS-CoV-2 has had a profound impact not only on the people infected, but also on the whole population, negatively affecting the global economy and resources, leading to depression, anxiety, and other mental disorders [4,5]. Thus, the development of vaccines has been urgent for limiting SARS-CoV-2 transmission, with the aim of reaching a state of so-called herd immunity.

As of 1 March, there are five vaccines in phase IV trials—Pfizer/BioNTech + Fosun Pharma (BNT162b2); Moderna + National Institute of Allergic and Infectious diseases (mRNA-1273); AstraZeneca + University of Oxford (AZD1222); Sinovac Research and Development Co. (CoronaVac); and Janssen Pharmaceutical (Ad26.COV2.S, by Johnson and Johnson)—and more than 200 preclinical and clinical candidates. This has been made possible by coordinated work between the scientific and medical communities and governmental agencies, such as the Food and Drug Administration (FDA), to develop and approve effective vaccines in record time [6].

In the present review, the latest knowledge about vaccines against COVID-19 is addressed, along with a reminder of the importance of vaccines as an effective means of controlling communicable diseases and ensuring successful strategies in pandemic times. A brief summary of the characteristics and pathogenesis of SARS-CoV-2 is also provided, considering the present and future challenges of vaccination in the management of this pandemic.

## 2. Structure and Pathogenesis of SARS-CoV-2

SARS-CoV-2 is a member of the *Coronaviridae* family of enveloped and positive-sense single-strand RNA viruses [7]. Its genome comprises 29,903 nucleotides, divided, from 3´to 5´, into open reading frames (ORFs) 1a and 1b, encoding non-structural proteins. Then, it appears to encode conserved structural proteins: the spike (S protein), envelope (E protein), membrane (M protein), and nucleocapsid (N protein) proteins, along with several accessory proteins (3a, 6, 7a, 7b, 8, and 10) [8,9]. Like the genomes of many RNA viruses, the SARS-CoV-2 genome is continuously mutating, particularly due to the virus’ high rate of replication [10]. Thus, different SARS-CoV-2 strains have been described since the beginning of the pandemic, such as the so-called UK, South African, and Brazilian variants, which have exhibited increased transmissibility and infectivity [11,12,13]. SARS-CoV-2 binds to the cell receptor ACE-2 through the receptor binding domain (RBD) present in the viral S protein [14]. Transmembrane protease serine 2 (TMPRSS2) and cathepsin B/L are also key regulators of viral entry located at the plasma membrane and endosomal compartments, respectively [15]. Upon entry, viral RNA is released, and ORF1a/b is translated by cell ribosomes into non-structural and replicase proteins, such as RNA-dependent RNA polymerase (RdRp), a key enzyme for the replication and transcription of the virus [16]. The replication process is conducted in double-membrane vesicles, derived from the endoplasmic reticulum, and single strand(ss)-positive RNA is used as a template for the ss-negative and subgenomic RNA translation of both structural and accessory proteins. Finally, new virions are assembled, transported to the cell membrane, and released by exocytosis [17,18]. It is important to understand that ACE-2 is widely expressed in human tissues, including the lungs, brain, gastrointestinal tract, blood vessels, and skin [19]. Thus, SARS-CoV-2 is able to infect almost every cell type in the human body, although the respiratory tract is the most affected organ in severe cases of COVID-19, leading to acute lung injury or pneumonia, frequently associated with coagulopathies [20,21].

Every person is susceptible to SARS-CoV-2 infection, but certain groups are more likely to suffer from severe presentations of the disease. These groups include older people [22], patients with comorbidities and non-communicable diseases [23,24], and also pregnant women [25,26]. They all share an altered immune status, which is key to fully understanding SARS-CoV-2’s pathogenesis. It is important to characterize the innate immune response, the first line of defense provided by macrophages and granulocytes, which acts in coordination with adaptive immune cells T and B lymphocytes and concludes with the production of antibodies or immunoglobulins (Ig) that are specific against SARS-CoV-2 [27]. Previous studies have observed that IgM and IgG appeared 15 days after infection, with detectable seroconversion 3–4 weeks after symptom onset [28]. Importantly, IgA appears earlier and is more prolonged than IgG in its persistence [29]. Importantly, the Ig levels in some mild cases can be low or undetectable, which leaves the potential for re-infections [30]. The S protein is the major target of human Ig, hence inducing SARS-CoV-2 neutralization [31], whereas Igs against the N protein may also be produced, although without neutralization [32]. However, if the immune response fails, SARS-CoV-2 may be able to disseminate throughout the organism, promoting an aberrant immune response with uncontrolled cytokine production (a so-called “cytokine storm”), acute respiratory-distress syndrome (ARDS), and multiorgan failure [33]. In fact, immune cells, as well as inflammatory components, have been proven to be valuable markers for predicting COVID-19 fatality [34]. Therefore, it is crucial to ensure an adequate immune response to prevent or mitigate SARS-CoV-2 infection. During the last year, different therapeutic approaches to limiting the severe damage caused by COVID-19 have been studied. For instance, protease inhibitors to block SARS-CoV-2 entry, polyprotein maturation, or viral assembly; nucleoside analogues (e.g., remdesivir and ribavirin) to limit viral replication; chloroquine/hydroxychloroquine; or specific compounds targeting the immune system, such as interleukin-6 (IL-6) inhibitors and corticosteroids, are some examples of proposed therapies for combating SARS-CoV-2 infection [35,36]. Notwithstanding the efforts made in finding an effective treatment, there are still no approved therapies for COVID-19 and, although much attention was initially given to finding a potential cure, the lack of scientific evidence supporting the use of proposed treatments has shifted the focus to alternatives. In this context, vaccination could play a central role in this pandemic, limiting SARS-CoV-2 transmission, and COVID-19 cases. Furthermore, vaccination may provide countless benefits for the global situation on multiple levels, as discussed below.

## 3. Global Perspectives about the COVID-19 Vaccine and the Importance of Vaccination in the Pandemic

This is not the first time that a pandemic has occurred. Throughout history, multiple infectious agents have emerged with devastating consequences for humankind. The Black Death, the Spanish flu, SARS, Ebola, and Zika are just some examples of prominent pandemics in the past [37]. Vaccination undoubtedly represents a powerful tool for limiting the progression and dissemination of infectious agents. As an important precedent, vaccination prevented the spread of a smallpox outbreak in Yugoslavia in 1972 [38]. In 2009–2010, during the swine flu pandemic, a novel vaccine was developed four and a half months after its beginning. The collected data show that only four out of 30 European countries were satisfied with their vaccination rates, as different surveys reported that people were not willing to accept a vaccine with unproven safety regarding the risk of being infected by this H1N1 virus [39]. This is partly due to a central concern regarding vaccination, known as vaccine hesitancy. Even in the area of child vaccination, there are some parents who are still not convinced of the necessity of vaccines and their safety [40]. Notwithstanding the fact that vaccines are widely viewed as a symbol of success in human history, it is also true that vaccination has led to unexpected problems, particularly during the 20th century. However, the errors that occurred in the past resulted in even more caution being taken in the approval of vaccines [41]. In this sense, and in order to be objective, scientific inquiries must support the acquisition of further knowledge regarding vaccination. Although a causal relationship may be established between certain undesired events and the uptake of any type of vaccine, the evidence has shown us that these adverse effects are very rare and the overall safety profiles of vaccines are excellent, with vaccines playing a key role in preventing infections and protecting against infectious agents worldwide [42,43].

In the case of the SARS-CoV-2 pandemic, the urgent need for a vaccine is evident, as recently it has greatly exceeded the H1N1 influenza virus, as well as the SARS and MERS coronaviruses in terms of the total cases and deaths worldwide [44], with a sustained and alarming rate of new infections and deaths each day. Thus, it is essential to encourage citizens to get vaccinated to slow down SARS-CoV-2 transmission and the deaths caused by this novel pathogen. In this context, the purpose of vaccination is to reach a state of herd immunity. This term refers to an epidemiological state where a sufficient number of individuals have developed immunity against the pathogen in such a way that the rate of transmission between infected and susceptible people is notably reduced [45]. In the case of SARS-CoV-2, considering its properties, the estimated herd immunity threshold is 67%. In other words, the incidence of infections and transmission will not be reduced unless at least 67% of individuals develop SARS-CoV-2 immunity [46]. In this line, there are two routes to herd immunity: people get infected or people getting vaccinated. The second is undeniably the better option, as the potential damage and dangers from SARS-CoV-2 infection would be avoided. Vaccination represents a greatly effective method for achieving host immunization, leading to a coordinated response in either innate or adaptive immunity, as well as an immunological memory in both systems [47]. In this line, Self et al. [48] reported a significant decline in the presence of Igs approximately 2 months post-infection, but an initial higher detection ensured increased Igs after this period. This is what vaccination aims to provide—such a sustained, effective Ig response. In the same manner, inducing a proper T and B cell response represents a central action of vaccines, as their protective roles have been well-described in various animal models [49]. Vaccines are playing a key role in the SARS-CoV-2 pandemic, and they will be crucial to prevent a possible SARS-CoV-2 resurgence during the coming years [50]. Regardless of the fact that there are still many unknowns regarding the efficacy of the SARS-CoV-2 immune response, Ig production, or their durations, encouraging data show that Ig may remain detectable even 3 years post-infection for SARS-CoV [51,52], demonstrating that it may be possible to achieve a lengthy immunization. These data provide a ray of hope in terms of the possible effects that a vaccine may provide for SARS-CoV-2 immunization, although they ought to be taken with caution.

Social attitudes are a key issue for achieving herd immunity. It is also important to mention that, to guarantee a successful and effective vaccination program, governments and social media should encourage citizens to be willing to work together to solve the public health problem [53,54], as well as providing information to relieve their uncertainties. In several countries, survey studies have noted varying vaccine acceptance rates, so hesitancy needs to be addressed at the beginning of vaccination programs [55]. The countries with the highest rates of acceptance are Vietnam (98%), India (91%), China (91%), Denmark (87%), and South Korea (87%), whereas Serbia (38%), Croatia (41%), France (44%), Lebanon (44%), and Paraguay (51%) are those with the lowest acceptance rates [56]. Other countries, such as Tanzania, have refused SARS-CoV-2 vaccinations because, in the words of their Health minister, they are not “yet satisfied that those vaccines have been clinically proven safe” [57]. Similarly, the acceptance rate of vaccination also varies among different groups or professions. For instance, university students and healthcare workers dealing with COVID-19 patients, who are more conscious of the global problem, are more predisposed to getting the vaccine [58,59], hence demonstrating the importance of creating awareness among the general population.

In the same manner, during the third wave, an increase in the doubts regarding vaccination was observed in comparison with the first wave surveys [60]. This is probably related to another central issue in vaccine hesitation: the question of how the vaccines have been developed so promptly. It is true that it is an unprecedented situation, and vaccines normally take longer to be approved, even years. However, various factors must be considered. During the 21st century, vaccinology has experienced exponential growth, due to the emergence of current technologies. In this sense, COVID-19 has represented a global concern of great interest, to which many of these novel resources have been utilized [61,62]. A novel vaccine candidate is always tested following well-established criteria, involving pre-clinical (in vitro and in vivo studies) and clinical studies in humans (Phase I, Phase II and Phase III trials), as thoroughly described by Singh and Mehta [63]. All the COVID-19 vaccines have been subjected to and passed the different stages, proving their efficacy and safety. Conspiracy theories have grown in strength during the COVID-19 pandemic, leading to a wide plethora of misinformation that has negatively influenced vaccination acceptance [64]. Because of this, global, national, and regional institutions, along with experts in various fields, ought to supply scientific evidence to the general public with the purpose of educating them on vaccination and countering fake news or misunderstandings from anti-vaccine sources [65,66,67].

**Figure 1:**
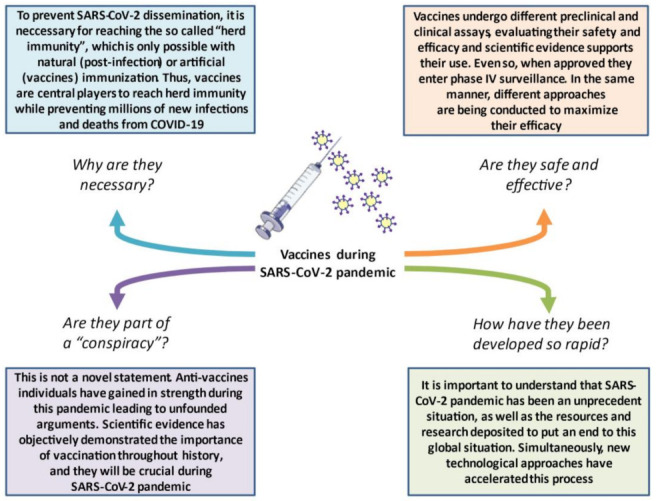
The role of vaccines during the SARS-CoV-2 pandemic and the main concerns in the general population. The proper communication and exposition of the main concerns regarding vaccination are crucial for ending this global predicament.

Overall, it is essential to understand that, in the COVID-19 pandemic, not only do human lives have to be considered, but the economy and quality of life are also central variables in the pursuit of timely herd immunity. Thus, it is essential to understand COVID-19 vaccines and communicate their importance to the global population, as summarized in Figure 1.

An inconvenient fact is that there are no long-term studies yet available analyzing vaccine reactions or the progression of SARS-CoV-2 infections in vaccinated individuals. However, the benefits of vaccinating the whole population exceed the risks because returning to normalcy as soon as possible represents a major need.

In the following sections, the approved vaccines, and some strategies recommended and followed by some countries, are reviewed.

## 4. Strategies and Considerations in COVID-19 Vaccination Programs

The distribution of vaccines and the question of how they are administered are also central concerns regarding COVID-19 vaccination. People are being classified according to priority to receive the vaccination. First, this is key to assuring a fair and equal distribution of vaccines globally, to provide an effective response against the SARS-CoV-2 pandemic [56]. It is critical to integrate many perspectives to create an effective vaccination campaign, including socioeconomic, immunological, and clinical factors, while also addressing vaccine hesitancy and politicization [68]. The target population for COVID-19 vaccination should be classified according to three central purposes: (1) maintaining essential societal services, (2) reducing severe COVID-19 presentations, and (3) limiting symptomatic infections and virus dissemination [69]. The first group are represented not only by healthcare workers but also by professionals tasked with maintaining public order (police and military), transport staff, teachers, food service workers, and, in general, any workers in indispensable jobs fulfilling core functions in our society [70]. The second group is composed of vulnerable populations, mainly older people, patients with previous comorbidities, and pregnant women, as previously stated. Here, it is vital to unravel the role of aging in COVID-19, as it is closely related with immune dysfunction (e.g., immunosenescence and inflammaging), contributing to worse SARS-CoV-2 severity [71]. Aging is a complex process in which many factors far beyond genetics are involved, such as epigenetics and lifestyle [72], which are equally important for distinguishing between healthy and pathological aged people. In this line, biological rather than chronological age represents a much better predictor of aging [73], and this should be considered when identifying the vulnerable aged population. Furthermore, another group that is seriously affected by the SARS-CoV-2 pandemic is racial and ethnic minorities. The impact of COVID-19 in this population can be understood not only from a biological perspective but also regarding social or economic factors, with certain communities being more predisposed to suffering from poorer outcomes [74]. This is a critical issue to address, as a recent study found that only 25 out of 219 clinical trials have taken place in lower-middle or low-income countries, and up to 80% of the people recruited in active vaccination trials were not from ethnic minorities, leading to an underrepresentation of these populations [75]. Thus, it is imperative to also include these populations as a priority group for vaccination.

Finally, the third group is composed of younger people with no underlying conditions, and according to the risk of virus dissemination and economic factors, they could be classified into three groups: (a) people aged between 20 and 59 years, (b) those aged 5 to 19 years, and (c) children ranging from 0 to 4 years old [69]. The last two groups are expected to be the last groups to be vaccinated globally. It is true that, generally, children and adolescents are less affected by SARS-CoV-2, and vaccines should demonstrate strong safety and efficacy before initiating global vaccination in this group [76]. Nevertheless, a vaccine targeting this population will be crucial for preventing SARS-CoV-2 transmission in the whole of society, while also avoiding possible severe COVID-19 presentations in both children and adolescents [77,78,79]. In the case of newborns, the vaccination of pregnant women could be an interesting approach, as SARS-CoV-2 antibodies may be detected in breastmilk [80]. Asymptomatic patients or people who have recovered from COVID-19 generating null or poor immune responses and even people who received first-generation COVID-19 vaccines with suboptimal immune responses should also be considered as potential targets for vaccines [81].

According to the Center for Disease Control and Prevention (CDC) recommendations [82], an adequate distribution of vaccines will include the following: Phase 1a—the vaccination of healthcare personnel and residents of long-term care; Phase 1b—frontline essential workers and people aged ≥75 years old; and Phase 1c—people aged 65–74 years or 16–64 years with underlying medical conditions and other essential workers. Afterwards, when the doses of the vaccines available increase, other groups will be included. Nonetheless, experts have suggested that it will require 6 months to a year to distribute COVID-19 vaccines to the general population, entailing various difficult situations to handle [83]. The United Kingdom and Israel are the leading countries in COVID-19 vaccination. Different factors have contributed to this accomplishment, particularly, robust and coordinated efforts within healthcare systems and governmental institutions [84,85]. Some examples of these factors are the stratification of fewer groups with a reduced age threshold to be prioritized, their rapidness in starting with massive vaccination programs, and the creation of multiple vaccination sites [86,87,88]. However, this does not necessarily equate to success. There is still a difficult road ahead before the end of the pandemic. During this period, it will be crucial to ensure the adequate monitoring and follow-up of COVID-19 vaccine recipients. According to the data collected by the WHO, almost 400 million vaccine doses have been administered worldwide [2]. This represents a great number of people getting vaccinated globally. In addition, as previously mentioned, this includes some vulnerable groups. Thus, it is not unlikely that, during these months, some people who received the COVID-19 vaccine may present with adverse outcomes, which could be attributable (or not attributable) to the vaccines (correlation does not imply causation). We must abide by objective and measurable scientific criteria. Further studies are also needed to evaluate re-infection rates and the duration of SARS-CoV-2 immunization after vaccination in the general population and vulnerable populations. A recent study conducted in 2020 by Hansen et al. [89] showed that there was no evidence of waning protection over time (3–6 months vs. ≥7 months). However, they found significant differences in the natural immunization of people aged ≥65 years (less than 50%) in comparison to that for younger people (80%). In this sense, it would be particularly important to monitor older people after vaccination to observe the effects of vaccines in this particular group. The outbreak of novel SARS-CoV-2 variants is equally a core issue that must be addressed simultaneously with vaccination. A dynamic model and statistical analysis conducted by Davies et al. [90] estimated that the UK variant could lead to higher rates of COVID-19 hospitalization and deaths in 2021 than those that occurred in 2020. Hence, monitoring the effectiveness of vaccination and the protection provided by vaccination against these strains in the general population will be equally important in the global management of the pandemic.

Overall, a coordinated regional, national, and international approach is needed to obtain the maximum benefits of vaccination, while also aiding to develop effective strategies and steps to reach herd immunity.

## 5. Vaccines in Clinical Development

Since the pandemic was declared a year ago, searching for a vaccine has become the top priority [91]. The time required was much shorter than what is usually observed with the discovery of vaccines or drugs. The whole world was anticipating the development of a compound, and high investment, biotechnology and logistics were the main drivers in the development process.

All the data related to the candidate vaccines in development have been gathered and summarized in WHO publications. The data collected to date show 82 candidates in clinical development and 182 in pre-clinical phases. Research is ongoing to make more vaccine variants available and to determine for how long the vaccines that have already been distributed will protect against COVID-19. There are currently four candidates that the FDA has authorized for use urgently and that have, hence, advanced to Phase IV. Three of these have already been widely distributed during the last two months (Pfizer/BioNTech + Fosun Pharma; Moderna + National Institute of Allergy and Infectious Diseases; and AstraZeneca + University of Oxford). Recently, the other two (Sinovac Research and Development Co. and Janssen Pharmaceutical by Johnson and Johnson) have just been added to the list of the first COVID-19 vaccines [92]. Once in Phase IV, COVID-19 vaccines are subject to pharmacovigilance, which is conducted by local and international institutions, working actively and in coordination.

Conventional vaccines are based on attenuated or inactivated viruses. In the case of SARS-CoV-2, this was not easy, as the whole virus contains elements that can provoke disease in vulnerable patients. This required previous knowledge on the attenuation of RNA viruses and, specifically, on coronaviruses with exonucleases, to avoid reversion to virulence [93]. Later preliminary data for SARS-CoV concerning the use of spike proteins as a possible target helped to advance the development of vaccines against SARS-CoV-2 [94]. In this area, immunoinformatics provided several multi-epitope designs in silico [95]. The desire was to make the immune system encounter (preferably) a fragment of the virus and unleash a specific recognition response. To trigger a rapid and effective adaptive immune response, mRNA vaccines (Pfizer/BioNTech and Moderna), with higher reported efficacy, were perfect candidates, as their mechanism consists of using the capacity of the individual to translate the encoding mRNA to specific SARS-CoV-2 antigens. By contrast, the other vaccines in clinical development are viral-vector-based (AstraZeneca + Oxford and the more recent Janssen Pharmaceutical vaccine) and, more recently, inactivated-virus-based (Sinovac), but these have also shown promising results. The success of those vaccines that advanced more quickly through clinical trials was largely due to well-designed routes of administration and immune-response studies [96].

The first vaccines to reach Phase IV (Pfizer/BioNTech, Moderna and AstraZeneca + Oxford) involve two-dose regimens of the vaccine formulae. In fact, most of the candidate vaccines (62% to date) involve this number of doses, with 2 to 3 weeks between each shot. Furthermore, most of the routes of administration are intramuscular (IM) (77%), which is the case for the top three, whereas subcutaneous or intradermal routes are in the minority [92]. Further research is still needed to determine optimal dose regimens and minimize costs, along with minimizing adverse events and maximizing protection [97].

**Figure 2:**
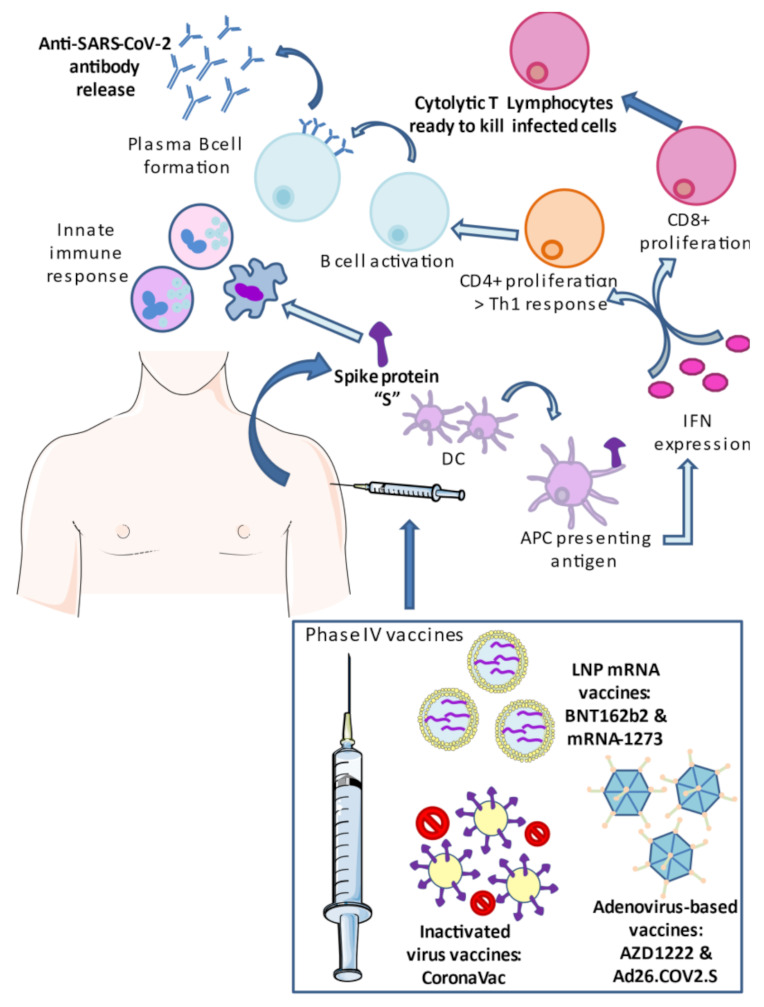
Designs of, and immune reactions promoted by, COVID-19 vaccines. mRNA-based, adenoviral vectors, or inactivated virus can induce a coordinated innate and adaptive response, prominently limiting the damage caused by SARS-CoV-2 infection. In all cases, the recognition of the spike protein activates the innate immune system and interferon pathways, showing antiviral properties, promoting T and B cell responses, with an augmentation of cytolytic T cells and antibodies secreted against SARS-CoV-2.

To date, all the vaccines authorized for administration to the general population have shown seroconversion (a change from a seronegative state at baseline to a seropositive state with administered doses). The mechanisms of the main platforms that have been already distributed worldwide—namely, mRNA-based, adenovirus-based, and inactivated viral vaccines—are summarized in Figure 2 and are briefly addressed below, together with safety and efficacy issues.

### 5.1. mRNA-Based Vaccines

mRNA vaccines are first-in-class tools in the biotechnological sector, promising a fast solution. The mRNA molecule is rigorously modified to avoid undesired reactogenicity and delivered via lipid nanoparticle systems to favor successful delivery, which has contributed greatly to the success of SARS-CoV-2 vaccines in particular. This was the case for the Pfizer/BioNTech (BNT162b2) and Moderna (mRNA-1273) vaccines, which showed more than 94% efficacy in Phase III clinical trials and were the first two vaccines distributed worldwide. These two vaccines contain the sequence encoding the spike (S) proteins.

Previous studies on mRNA vaccines have shed light on SARS-CoV-2 vaccine development. A few years ago, biotechnological improvements suggested that these vaccines were potential modulators of the innate and adaptive immune response, as well as being stimulators of antigen-specific T cell responses. These data were correctly demonstrated and collected from animal models, with the first work showing that in vivo gene injection, in the transcriptional form, could result in the encoded protein actually being produced [98]. This technology was used in clinical trials for infectious diseases and some types of cancer. Some reviews reported the ability of mRNA vaccines to elicit a cellular and humoral response with no interaction with the host genome [99]. Preclinical in vitro research on the influenza A virus showed the advantage of optimizing the mRNA sequence with a view to new strain outbreaks [100], which is also applicable to SARS-CoV-2. Moreover, the strengths of this type of vaccine platform include the fact that it is easily scalable and its low cost of production, making it possible for the supply to meet the demand derived from the pandemic situation [101]. Additionally, mRNA technology is also being studied for expressing monoclonal antibodies for passive immunotherapy [102] that could be used in patients with severe clinical manifestations. This technology requires more research related to the immunological response, construction, or delivery using nanotechnological elements than the rest of the vaccine candidates.

#### 5.1.1. Delivery

The delivery of mRNA in non-toxic carriers was the main challenge for guaranteeing its effect, because mRNA is a very labile and easily degradable molecule, particularly given the widespread presence of RNases [103]. Cutting-edge nanotechnology research suggested the use of lipid nanoparticles (LNPs) as formulations that facilitate the release of mRNA into cells from endosomes [104,105]. The design for BNT162b2 and mRNA-1273 included low copy numbers (1–10) of mRNA bathed in aqueous solution and attached to the LNP, which consists of a bilayer lipidic vesicle with polyethylene glycol (PEG) on the surface and cholesterol, phospholipids, and an ionizable lipid in the structure. Each design used a different ionizable lipid, resulting in differences in thermostability. This central component is key for the release of mRNA molecules after IM injection, with a neutral charge at physiological pH but protonated form in the endosome at a lower pH [106].

The formulation of these LNPs allows not only the delivery of mRNAs but also an adjuvant effect itself [107]. LNPs codeliver adjuvants to enhance the immune response specifically acting on antigen-presenting cells (APCs). Moderna affirms that their candidate uses the LNP as an adjuvant based on the properties of its structural lipids, and BioNTech/Pfizer state that their RNA is the adjuvant for eliciting TLR-mediated activity [108].

More research continues in the field of the delivery of mRNA vaccines, suggesting that, along with liposomes, other components could serve as coadjuvants. An example recently studied in mice is a conjugate of polyglucan and spermidine, with a positive charge, which aids in enveloping negatively charged mRNA [109].

#### 5.1.2. Immunological Response

Studies in animal models showed that, after intradermic injection, dendritic cells, acting as antigen-presenting cells (APCs), take up the mRNA and carry it to lymph nodes. There, the APCs activate adaptive immunity by presenting mRNA molecules to T cells and inducing their proliferation and activation [110]. Clinical trials in humans have been reviewed to reaffirm that free mRNA molecules released from the lipid nanoparticle are recognized by toll-like receptor 3 (TLR3), TLR7, TLR8, or retinoic acid-inducible gene (RIG-I), which activates type I interferon (IFN I) production and stimulates the Th1 response. On the one hand, ribosome translation is followed by proteasomal degradation, the peptides derived from which are presented by the major histocompatibility complex I (MHC-I), and, on the other hand, there is also MHC-II presentation resulting from APCs’ antigen uptake [111]. In lymph nodes, antigen presentation to T and B cells boosts the establishment of germinal centers, where antibody-producing cells are stimulated along with memory B cells [112]. In vaccinated participants, an increase in C-reactive protein was also observed, which is an indicator of vaccine adjuvant activity [107].

In the event of potential mutations reported to have higher infectivity [113,114], studies on mRNA-1273 have also evaluated its ability to boost the CD8+ T cell response and antibody responses that may neutralize cells with wild-type spike protein (D614) or D614G mutants [115].

mRNA vaccines show particular strength, as it has been observed that they can elicit not only humoral but also cellular adaptive responses, activating T-cell helpers (Th) and cytolytic T lymphocytes [116]. This is relevant, as some longitudinal studies have noted the rapid decline of anti-SARS-CoV-2 IgG in mild COVID-19 patients [117].

#### 5.1.3. Safety and Efficacy

In the case of the Pfizer/BioNTech vaccine (BNT162b2), a large sample of individuals (43,548) in trials demonstrated 95% effectivity in preventing COVID-19 and an efficacy between 90% and 100%, along with high safety, with very few adverse events, such as pain at the injection site, fatigue, or headaches [118]. The safety, tolerability, and immunogenicity of two candidates were evaluated in Phases I, II, and III of clinical trials (NCT04368728).

Phase I clinical trials for the Moderna vaccine (mRNA-1273) concluded with higher titers of antibodies and systemic adverse effects, including fatigue, chills, headaches, myalgia, and pain at the injection site, both with the second dose [119]. This vaccine was proven firstly in participants 18 to 55 years old [119] and also in adults older than 56 years, promoting Th1 responses in both groups [120]. The effectiveness is already being evaluated in citizens for both the BNT162b2 and mRNA-1273 vaccines (NCT04760132), and worldwide, the outcomes have been quite positive to date [121].

Although some rare allergic reactions have been encountered, it is too soon to determine if the liposome ingredients may be allergenic; nevertheless, vaccines may have this effect, so it is something that must be considered [122]. Contraindications and precautions for patients with known hypersensitivity to ingredients of the vaccine (e.g., PEG) must be listed and explained before administration [123], and treatments, such as epinephrine injections to ameliorate post-vaccination anaphylaxis, must be available according to practical guidelines [124]. Public alarm was raised when rare thrombocytopenic events were reported in response to both the Moderna and Pfizer vaccines, but the FDA judged the benefits of vaccination to outweigh the risks [125].

### 5.2. Adenovirus-Based Vaccines

In this case, the delivered genetic cargo is based on adenovirus vectors, which are non-replicating and can boost the immune response without the presence of adjuvants [126]. This is the case for the AstraZeneca + Oxford vaccine and the Jansen Pharmaceuticals vaccine by Johnson and Johnson, both of which encode the S protein. The first one uses a chimpanzee adenovirus vector (ChAdOx1-S-(AZD1222)); the latter (Ad26.COV2.S) relies on a recombinant human-based adenovirus vector. The Janssen vaccine has an additional benefit in comparison to the other candidates, as it is administered in only one dose, which reduces manufacturing costs. The Sputnik V or Gam-COVID-Vac-Adeno-based (rAd26-S + rAd5-S) vaccine by the Gamaleya Research Institute of Epidemiology and Microbiology, still in Phase III, is different to the previous three. This Russian model employs two different common cold adenovirus vectors (rAd26 and rAd5) instead of one [127].

This engineering approach has emerged in the last two decades, providing good vectors for vaccines and for gene therapy in cancer with oncolytic adenoviruses. The advantages in this case reside in the possibility of constructing a vaccine with more elements [128] or manipulating them to enhance their efficacy [129]. The idea of using this platform for infectious diseases appeared in 1984 [130], when monkey adenoviruses were used as carriers [131], and they were suggested to also be useful for coronavirus vaccines when the SARS outbreak occurred in 2002 [132]. Some trials in nonhuman primates with this type of vaccination demonstrated T cell responses and antibody responses against the spike protein of SARS-CoV [133]. Additionally, adenovirus vaccines seem to be easy to construct and purify to high titers, ensuring genetic stability and affordability [134].

#### 5.2.1. Immunological Response

This type of vector elicits effective immune responses and emulates the real infection through the transgene products expressed. The use of (non-replicating) viral vectors allows the signaling pathways of not only antibody expansion but also a robust cytotoxic T response, with the power to destroy infected cells [135]. The design of the vector includes epitopes that are expressed in the capsid and presented by APCs by means of MHC class I and II. The induction of memory T cells is due to the fact that most adults have been infected by several adenoviruses and, consequently, there is a reactivation of anti-adenovirus effector memory cells [134].

Some evidence generated from trials shows that immunization with AZD1222 provokes a rapid activation of both humoral and cellular responses, with higher rates of response and lower reactogenicity at the second dose, with the neutralizing antibody titers being dose-dependent [136].

On the contrary, rAd26-S + rAd5-S, applying one adenoviral vector per dose, was thought to avoid a possible cytotoxic reaction against an only-adenoviral vector in a second injection. Reports of Phase I and II trials evidenced humoral and cellular responses in all the participants [137].

#### 5.2.2. Safety and Efficacy

After Phases I and II (NCT04436276), the Janssen vaccine, Ad26.COV2.S, in Phase III trials involving 45,000 volunteers aged 18 years and older, showed 66% efficacy in general terms, but 85% efficacy in preventing severe disease or hospitalization in vulnerable patients 28 days after IM administration (NCT04505722) [138]. The safety, efficacy, and immunogenicity of AZD1222 were also assessed, with acceptable outcomes and tolerability [136], in this case using 32,459 participants aged 18 years and older in Phase III (NCT04516746). Both candidates, along with the previous ones, have been enrolled in Phase IV trials with the authorization of the FDA (NCT04760132).

Although, in two months, it has not been possible to determine whether there are adverse effects in the long term, after administration, the most frequent events were pain at the injection site, myalgia, headaches, and fatigue. Two weeks after administering Ad26.COV2.S, a Th1 CD4+ response was detected in 83% of participants aged 18 to 55 years old and in 67% of participants older than 65 years [138].

The results from Phase I and II trials of Sputnik V show an optimal safety profile [137], and Phase III concluded with a 91.6% vaccine efficacy, which is higher than that of the three above-mentioned vaccines (NCT04530396), and with grade 1 adverse reactions, similar to those for the rest of the candidates [139].

Unlike with mRNA vaccines, there have been no reported cases of allergy for adenovirus vaccines [140]. It is still too soon to observe long-term outcomes, but allergic reactions are common adverse events in vaccination, and there is no contraindication to administering the vaccines to patients with no history of allergic disease. Nevertheless, in March (2021), new reports about a possible link of the AstraZeneca vaccine to rare thromboembolic events stalled vaccination programs with this vaccine in several European countries. A few days after the established impediment, the European Medicines Agency (EMA) assured the public that the benefits outweigh the risks and that there was no causality evidenced. Therefore, those countries resumed the administration of AZD1222 with exceptions [141,142]. Similarly, a recent study has reported that a two-dose regimen of the ChAdOx1-S-(AZD1222) did not show protection against mild-to-moderate COVID-19 caused by the South African variant [143]. This is a worrisome issue, as different SARS-CoV-2 strains are emerging simultaneously with vaccination. Thus, the appearance of second-generation vaccines will be crucial in targeting all these variants.

### 5.3. Inactivated Virus Vaccines

A conventional type of vaccine that has been widely used for decades is the inactivated virus vaccine. The idea is to destroy the infectivity of the virus in order to make it safe, while maintaining immunogenicity with high-quality antigens to elicit an immune response [144]. There are some problems associated with the inactivation process that should be tested before establishing in vivo models—these problems include the formation of virus aggregates, protein crosslinking, denaturation, and degradation [145]. Therefore, quality control is crucial for guaranteeing virus inactivation without destroying epitopes. To achieve this goal, some chemicals have been formulated and used in different concentrations to react with nucleic acids for disrupting replication [146]. This type of vaccine formulation is used for the investigated formaldehyde-inactivated whole-virus SARS-CoV-2 vaccine (CoronaVac) developed by Sinovac, which is currently being assessed in Phase IV. Other candidates may also use the same inactivated virus for different strains. Pilot-scale production has been undertaken of the candidate PiCoVacc, noted in preclinical animal models to result in antibodies that are able to neutralize a broader spectrum of SARS-CoV-2 strains [147]. In mid-March 2021, the Sinopharm + China National Biotec Group Co + Wuhan and Beijing Institute of Biological Products vaccines made advances in clinical trials, with one of their two candidates already in Phase IV (NCT04510207 and NCT04612972).

The Sinovac vaccine uses alum adjuvants. Although its efficacy has been proven, the limitations of this type of adjuvant have normally been the requirement for several doses for protection and a polarization toward the Th2 response over Th1, but further evidence from ongoing trials with CoronaVac is still needed to determine if there are differences with the previous evidence on alum-adjuvanted vaccines [148].

#### 5.3.1. Immunological Response

The mechanism is triggered similarly as in the previous cases highlighted above. Monocyte activation promotes IFN expression, and this activates CD4+ T cells, boosting antibody secretion by B cell activation, along with CD8+ cells, promoting the killing of infected cells [96]. CoronaVac elicited 92.4% seroconversion in participants after two weeks and 97.4% after 4 weeks with second administration [149]. A high titer of antibodies was also observed 6 weeks after immunization [150].

#### 5.3.2. Safety and Efficacy

In Phase I and II trials, patients showed a low rate of adverse reactions and presented immunogenicity (ChiCTR2000031809). Some evidence from Phase III trials with CoronaVac (NCT04383574), administered to adults aged 60 years and older, has shown adverse reactions in 20% of participants, including injection site pain and mild-to-moderate symptoms (time frame: 28 days between each dose). Some serious adverse events (2%) were considered as not being related to vaccination [151]. The immunogenicity and safety in adults aged 18–59 was also assessed, with acceptable outcomes ([149], NCT04352608).

### 5.4. Other Vaccine Platforms Awaiting Approval

According to the *WHO Draft Landscape of COVID-19 Candidate Vaccines*, viral vector vaccines represent 15% of the candidates in clinical development, whereas RNA candidates represent 12%, and inactivated viruses represent 13%. Surprisingly, protein subunit platforms predominate, at 33%, and some of their candidates, which are ready for mass rollout, have published safety and efficacy results and reached Phase III. This is the case for NVX CoV-2373/PREVENT-19 (SARS-CoV-2 rS/Matrix M1-Adjuvant, or full-length recombinant SARS CoV-2 glycoprotein nanoparticle vaccine adjuvanted with Matrix M), developed by Novavax. NVX CoV-2373 is a recombinant nanoparticle vaccine based on the SARS-CoV-2 S protein in a baculovirus of *Spodoptera frugiperda* with site-directed mutagenesis [152]. The distinctive feature of this model resides in a Matrix-M1 adjuvant that is mixed with the S protein before injection. Owing to this adjuvant, Phase I and II reports show titers of antibody responses much higher than those in COVID-19 convalescent serum from people who had recovered from more severe COVID-19 [123]. Novavax also showed 60% efficacy against the South African variant in Phase IIb trials (NCT04533399) and is already in Phase III, demonstrating 89.3% efficacy in the UK (NCT04583995).

## 6. Conclusions and Future Directions

Vaccination is playing a central role in the SARS-CoV-2 pandemic, impeding new infections, while saving millions of lives. However, there is no denying the fact that it is only the starting point and there are still many issues to address, such as unraveling the virulence ability of SARS-CoV-2 and choosing suitable experimental models and designs for clinical trials, and more robust scientific evidence is needed to fully evaluate the safety and efficacy of COVID-19 vaccines [96]. Additionally, the global economy has greatly suffered during this pandemic, and vaccines should also aim to achieve their maximum benefits with the minimum dose, such as Johnson and Johnson’s vaccine. Meanwhile, new vaccines are being tested in preclinical and clinical trials, and a wide variety of technologies are aiding in the development of new methods of vaccination. For instance, bioinformatics and molecular approaches may serve to identify novel epitopes, hence leading to better vaccine design [153,154]. The emergence of novel COVID-19 strains is also a central concern to be considered for present and future vaccinations. In this sense, an interesting objective could be to achieve the production of “super antibodies”, Igs that are able to induce a potent immune response against a wide variety of pathogens [155]. In fact, previous research has detected super-antibodies against SARS-CoV-2 in a SARS-CoV-immunized patient [156], and some authors have suggested the possibility of future pan-coronavirus vaccines targeting the entire coronavirus family [157], although this design is still in its infancy.

Currently, novel strategies of vaccination are being explored, such as combining different types of COVID-19 vaccines. Through this approach, also known as heterologous vaccination, it is possible to elicit superior immunogenicity when compared to using homologous shots [158]. In this line, some in vivo models have shown that heterologous vaccination results in enhanced immune responses with boosted cytotoxic T cell and Th1 activation [159]. Thus, a clinical trial, named ComCOV (Comparing COVID-19 Vaccine Schedule Combinations), is underway [160] to assess the efficacy of heterologous vaccination and other vaccine strategies against SARS-CoV-2. The results will shed light on potential approaches regarding general COVID-19 vaccination.

Another essential point of study for future vaccines is related to their routes of administration. Almost all COVID-19 vaccines are delivered via the IM route, generating a systemic immune response. Nevertheless, some authors have explored the potential of developing a vaccine capable of inducing a response directly in the respiratory mucosa, emulating previous experiences, such as those with influenza or measles, which would probably be more effective in the early control or clearance of SARS-CoV-2 [81].

Simultaneously, and together with vaccination advances, it is essential to follow the measures and suggestions proposed by each country. In this line, the combined use of vaccines and non-pharmaceutical interventions are the best methods for preventing SARS-CoV-2 infection [161], as this slows the transmission of the virus, while leading to herd immunity. Lifestyle interventions, such as following a healthy diet, getting adequate rest, and performing physical activity, might additionally support maintaining a healthy immune system, which is crucial for a proper response against SARS-CoV-2 infection, reducing the risk of severe COVID-19 and hospitalization [162,163,164]. In the same manner, research on additional therapeutic agents, such as immunotherapy [165] or plant-derived compounds [166], could equally provide potential tools for the clinical management of severe COVID-19 patients, as it is necessary to approach this pandemic from an integrative perspective.

In conclusion, COVID-19 vaccines are now critical players in the global SARS-CoV-2 situation, with the aim of progressively reaching herd immunity. The main characteristics, benefits, and current knowledge of the available vaccines are presented in Table 1. Although there is still a need to optimize the costs of manufacturing, and neutralizing antibodies are dose-dependent, most candidates are showing positive outcomes and neutralizing antibodies conferring protection against the disease. Even though COVID-19 immune responses do not last long term, as the antibody titers tend to decline within two months, rapid and efficient vaccination programs are important for blocking transmission. People’s trust in scientific evidence will be key throughout the following months, and public authorities should always provide feasible and accessible data, as well as appealing to citizens’ sense of responsibility and the implications for society, in such a way that everybody wisely decides to get vaccinated, as it is, without doubt the best path to follow. Although more efforts are still needed and more issues remain to be dealt with, we are on the way towards putting an end to this devastating pandemic.

## Author Contributions

Conceptualization, C.G.-M., O.F.-M., C.B., J.A.D.L.-L., M.A.Á.-M. and M.A.O.; methodology, C.G.-M., O.F.-M., L.G.G., M.A.O. and A.A.; validation, M.Á.-M.; formal analysis, A.A.; investigation, C.G.-M., O.F.-M., C.B., D.T.-C., J.M., L.G.G., E.S., M.Á.-M., L.S.-T., A.M.G.-L., N.G.-H., A.A., J.B., J.A.D.L.-L., M.A.Á.-M. and M.A.O.; resources, J.B.; data curation, D.T.-C., J.M., E.S., M.Á.-M., L.S.-T., L.G.G., A.M.G.-L., N.G.-H., A.A., J.B. and J.A.D.L.-L.; writing—original draft preparation, C.G.-M., O.F.-M., C.B., D.T.-C., E.S., M.Á.-M., L.S.-T., A.M.G.-L., N.G.-H., J.B., J.A.D.L.-L., M.A.Á.-M. and M.A.O.; writing—review and editing, M.Á.-M., L.S.-T., N.G.-H. and M.A.O.; project administration, J.B.; funding acquisition, M.Á.-M. and J.B. All authors have read and agreed to the published version of the manuscript.

## Figures and Tables

**Table 1:** Summary of the main COVID-19 vaccines currently distributed worldwide. IM, intramuscular.

Name	Developers	Platform	Route of Administration	Main Mechanisms of Response	Doses	Pros	Cons	Trials
BNT162b2	Pfizer/BioNTech + Fosun Pharma	1–10 copies of mRNA bathing in aqueous solution, transported by lipid nanoparticles	IM	Expression of the viral S protein encoded in the mRNA and antigen presentation; induction of IFN I release, stimulating Th1 response, antibodies and memory T and B cells.	2	High efficacy (app. 95%); few adverse effects	Possible allergic reactions	NCT04368728 NCT04760132
mRNA-1273	Moderna + National Institute of Allergic and Infectious diseases		IM		2	Higher titers of antibodies. Easier to transport and store	Broader adverse effects; possible allergic reactions	
AZD122 (ChAdOx1-S)	AstraZeneca + University of Oxford	Adenoviral vector	IM	Emulation of viral infection; inducing expression of IFN, antibodies, and memory T and B cells, along with T CD8 activity	2	Rapid activation of both humoral and cellular responses, with higher rates and lower reactogenicity at a second dose. Non-allergenic	Reduced efficacy in comparison with mRNA-based vaccines.	NCT04516746 NCT04760132
Ad26.COV2 S	Janssen Pharmaceutical by Johnson & Johnson-		IM		1	One dose vaccine (rentability), non-allergenic		NCT04436276 NCT04505722
Sputnik V (rAd26-S + rAd5-S)	Gamaleya Research Institute of Epidemiology and Microbiology		IM		2	Applies a different adenoviral vector per dose, avoiding possible cytotoxic reaction against an only-adenoviral vector in a second dose		NCT04530396
CoronaVac	Sinovac Research and Development Co.	Inactivated SARS-CoV-2	IM	Similar to adenoviral vectors; induction of IFN, memory T and B cells, antibody production, and T CD8 activity	2	92.4% seroconversion in participants after two weeks and 97.4% after 4 weeks with second administration reported. High titer of antibodies even 6 weeks after immunization	Higher adverse reactions during Phase III in adults aged ≥ 60 years old (20%), mainly pain at the injection site and mild-to-moderate COVID-19 symptoms)	NCT04352608
PREVENT-19 (NVX CoV-2373)	Novavax	Protein subunit	IM	Enhanced humoral responses and B and T memory cells	2	Matrix-M adjuvants promote higher titers of antibodies than serum content from convalescent COVID-19 patients	Subunit vaccines require adjuvants to improve immunogenicity.	NCT04533399 NCT04583995

## Data Availability

Not applicable.
